# Research trends in post-stroke aphasia (2004–2024): a bibliometric and visualized analysis

**DOI:** 10.3389/fneur.2025.1588130

**Published:** 2025-06-25

**Authors:** Yufeng Peng, Kewei Peng, Yunfeng Ying, Luyao Li

**Affiliations:** ^1^Zhenhai Hospital of Traditional Chinese Medicine, Ningbo, China; ^2^The Rehabilitation Department of Wuhan No. 4 Hospital, Wuhan, China

**Keywords:** visualization analysis, research Frontiers, post-stroke aphasia (PSA), aphasia after stroke, bibliometrics

## Abstract

**Introduction:**

This study employs bibliometric analysis to systematically investigate the evolutionary trajectory and disciplinary dynamics of post-stroke aphasia mechanism research from 2004 to 2024.

**Methods:**

Through multidimensional examination of 3,492 publications from Web of Science Core Collection.

**Results:**

We identify paradigm shifts characterized by three distinct phases: initial reliance on neuroimaging for anatomical localization of language-area lesions, subsequent focus on white matter remodeling and neuromodulation techniques validating neural plasticity hypotheses, and recent advances in functional connectomics integrated with multimodal intervention strategies. International collaboration exhibits marked geographic disparities, with the United States, United Kingdom, and Australia forming the knowledge-production nucleus through leadership in neuromodulation innovation and brain network research. While China ranks among top contributors in publication volume, it confronts dual challenges of insufficient transnational cooperation and underdeveloped culturally-adapted assessment tools. Notably, a persistent technology-practice gap persists as a critical bottleneck - despite neuroimaging’s dominance in mechanistic studies, clinical integration of functional assessment tools remains suboptimal, and neuromodulation trials demonstrate attenuated effect sizes compared to preclinical models. Temporal analysis reveals research imbalance favoring acute-phase intervention studies over chronic-phase management research. Emerging technologies such as digital therapeutics exhibit limited research clustering.

**Discussion:**

Based on these findings, we propose a multidimensional framework integrating precision neuromodulation, cross-cycle rehabilitation pathways, and digital ecosystems, prioritizing multicenter brain network database development and dialect-adaptive assessment scales. This investigation provides empirical mapping of current research landscapes and actionable insights for future investigations in post-stroke aphasia rehabilitation.

## Introduction

Post-stroke aphasia (PSA) represents one of the most common neurological impairments following cerebrovascular accidents, affecting 21 to 38 percent of stroke survivors (1). This acquired communication disorder significantly disrupts both speech generation and language comprehension while inducing substantial quality-of-life deterioration through social disengagement and vocational limitations (2). Clinical investigations demonstrate that variations in post-stroke aphasia manifestations originate from neuroanatomical differences within the left hemisphere’s language-specialized zones (3). The severity of damage to essential linguistic cortices, particularly Broca’s and Wernicke’s areas, combined with the spatial configuration of associated white matter pathways including the superior longitudinal fasciculus and arcuate fasciculus (4), serves as the principal determinant for classifying aphasia subtypes into fluent and non-fluent categories while simultaneously influencing functional recovery trajectories (5).

The methodological paradigm in PSA mechanism research has undergone substantial evolution over two decades. Initial investigations employed behavioral assessments combined with structural neuroimaging to analyze static structure–function correlations (6). With the advent of functional MRI and diffusion tensor imaging, research focus shifted toward dynamic language network reorganization mechanisms (7). Longitudinal data demonstrate that acute-phase hypoperfusion in left-hemisphere language areas strongly predicts initial deficit severity (8), while subacute compensatory activation of right-hemisphere homologs emerges as a critical neural marker for functional recovery (9). This bilateral hemispheric synergy framework provides novel theoretical insights into neuroplasticity-driven language reorganization (10).

Despite significant progress, critical knowledge gaps persist regarding the temporal dynamics of language network reorganization and their correlation with individual prognostic divergence (11). The substantial variability in neuromodulation treatment efficacy, particularly evident in transcranial magnetic stimulation protocols, underscores the urgent demand for biomarker-driven intervention frameworks. These challenges become further exacerbated by persistent translational inefficiencies stemming from inadequate cross-disciplinary collaboration between basic neuroscience, neuroengineering, and clinical rehabilitation domains (12, 13). Resolving these interconnected issues mandates comprehensive analysis of disciplinary evolution patterns and strategic integration of methodological advancements (14).

Bibliometric analysis, as a core scientific mapping tool, offers unique methodological advantages for delineating PSA research evolution (15). This study integrates CiteSpace’s burst detection, VOSviewer’s co-occurrence network analysis, and Bibliometrix’s temporal trend tracking to systematically reveal thematic evolution patterns in PSA mechanism research (16, 17). We identify critical technological drivers, quantify structural characteristics of international collaboration networks, and assess their disciplinary impacts (18, 19). The study included the literature on post-stroke aphasia from 2004 to 2024. Preliminary literature review indicated that while publications existed between 2000 and 2004, a significant acceleration in research output was not evident during this initial period. However, around 2004, the field witnessed the increasing application of neuroimaging techniques and non-invasive brain stimulation methods, concurrent with the maturation of relevant research paradigms. Consequently, research productivity in the PSA domain began demonstrating a significant growth trend from approximately this time point. Therefore, the 21-year timeframe of 2004–2024 was selected as it effectively captures the key developmental phases of the field, encompassing its initial advancements, subsequent rapid expansion, and the formation of current research hotspots. Within this context, the research status, latest progress and frontier research hotspots in this field were reviewed, and new research directions were pointed out.

## Methods

### Search strategy

**Figure 1:**
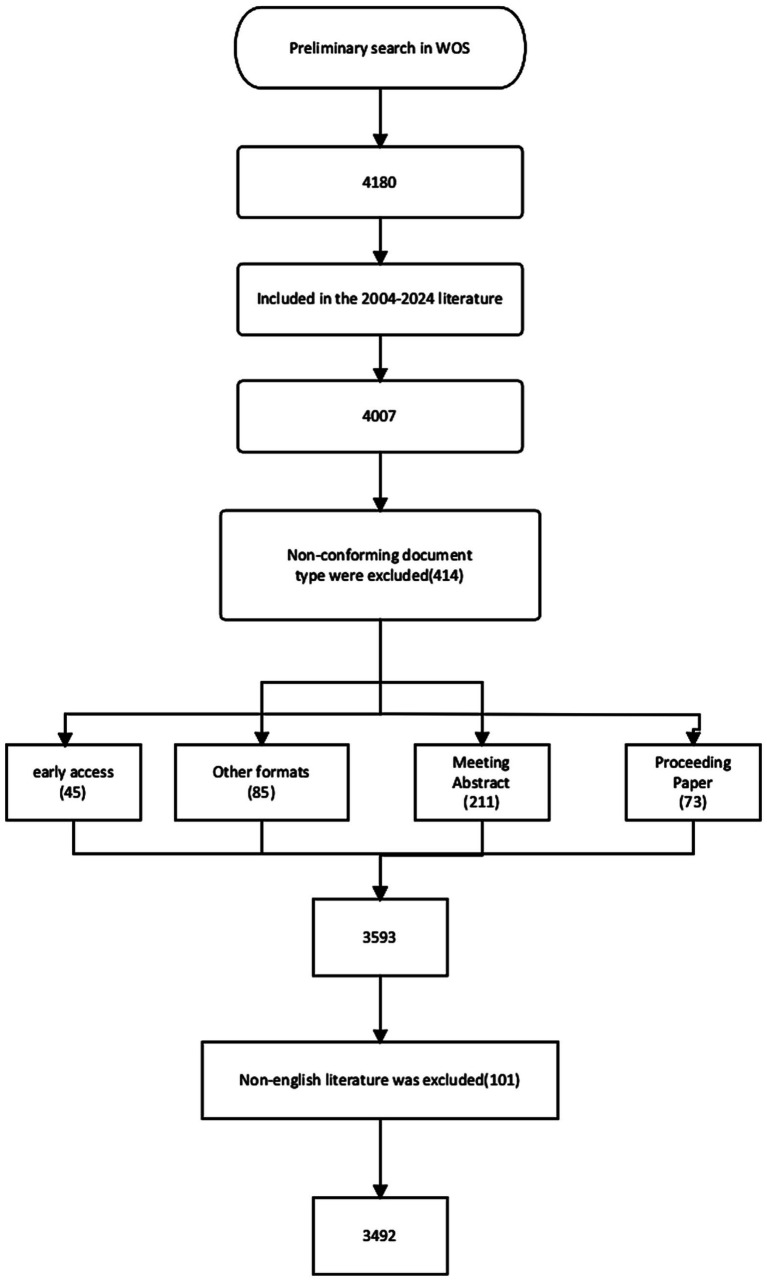
Flowchart of the study.

The Web of Science Core Collection (WoSCC), a globally recognized authoritative database, has emerged as the preferred database for bibliometric research. Using the Science Citation Index Expanded (SCIE) within WoSCC as the primary data source, this study compiled literature on post-stroke aphasia published between January 1, 2004 and December 31, 2024. The retrieved dataset includes comprehensive citation metadata essential for knowledge graph analysis, encompassing article titles, abstracts, keywords, author affiliations, institutional/country distributions, citation frequencies, and collaboration networks. Within the NIH National Library of Medicine database, we identified 28 terms using the search term ‘Stroke’ and 69 terms using ‘Aphasia’. Additionally, the subject headings ‘post-stroke aphasia’ and ‘aphasia after stroke’ were included. Based on the MeSH vocabulary, associated entry terms, and our research objectives, a comprehensive search strategy was established, which can be found in Supplementary material 1. Non-research publications such as editorial materials, conference abstracts, Early Access records, notes, book chapters, letters, retractions, and corrections were systematically excluded. Through rigorous full-text screening and abstract evaluation, 688 irrelevant records were removed, yielding a final analytical corpus of 3,492 publications. See Figure 1 for the flow chart.

### Data processing and analysis

Data processing and analysis involved extracting, cleaning, and standardizing the dataset from Web of Science Core Collection (WoSCC), which included “complete records and cited references” downloaded as plain text files. Raw data were directly imported into bibliometric software without format conversion, adhering strictly to PRISMA guidelines (20). Following retrieval, two independent reviewers (Yufeng Peng and Kewei Peng) screened titles and abstracts, with exported data labeled as “download_xxx.txt” containing metadata such as titles, publication years, authors, affiliations, keywords, abstracts, and journal information. Three complementary bibliometric tools—VOSviewer 1.6.2, CiteSpace 6.1.R, and Bibliometrix 4.1—were employed to analyze 21 years of post-stroke aphasia research. CiteSpace, developed by Professor Chaomei Chen, enabled visualization of citation networks and emerging trends through progressive knowledge domain mapping (17). VOSviewer optimized large-scale data interpretation by constructing interpretable bibliometric maps, while Bibliometrix, an R-based platform, facilitated scientific mapping using the “bibliometrix” package (21). These tools synergistically identified high-impact publications, collaboration patterns, and conceptual clusters, ensuring rigorous analysis of temporal trends and knowledge structures.

### Visualization analysis

The multi-dimensional analytical framework encompassed author networks, national/ institutional distributions, journal patterns, citation linkages, and keyword evolution. VOSviewer 1.6.2 facilitated co-occurrence and clustering analyses of institutions and authors, where node size reflected element prominence and color-coded clusters indicated conceptual groupings. CiteSpace 6.1.R enabled dual-map overlays of journal distributions, timeline visualization of citation bursts, and keyword co-occurrence clustering, with cluster labels derived from title terms, keywords, and abstract semantics of representative publications. Bibliometrix 4.1 generated temporal publication trends for institutions/authors and keyword heatmaps reflecting conceptual intensity. Journal impact factors were extracted from the 2024 Journal Citation Reports (JCR) in WoSCC, while SCImago Graphica 1.0.48 and Origin 2024 enhanced graphical representation and statistical validation of spatial–temporal patterns. This integrated approach systematically decoded collaborative networks, knowledge diffusion pathways, and disciplinary convergence characteristics across spatiotemporal dimensions.

## Results

### Annual publication volume, citation volume and trends

**Figure 2:**
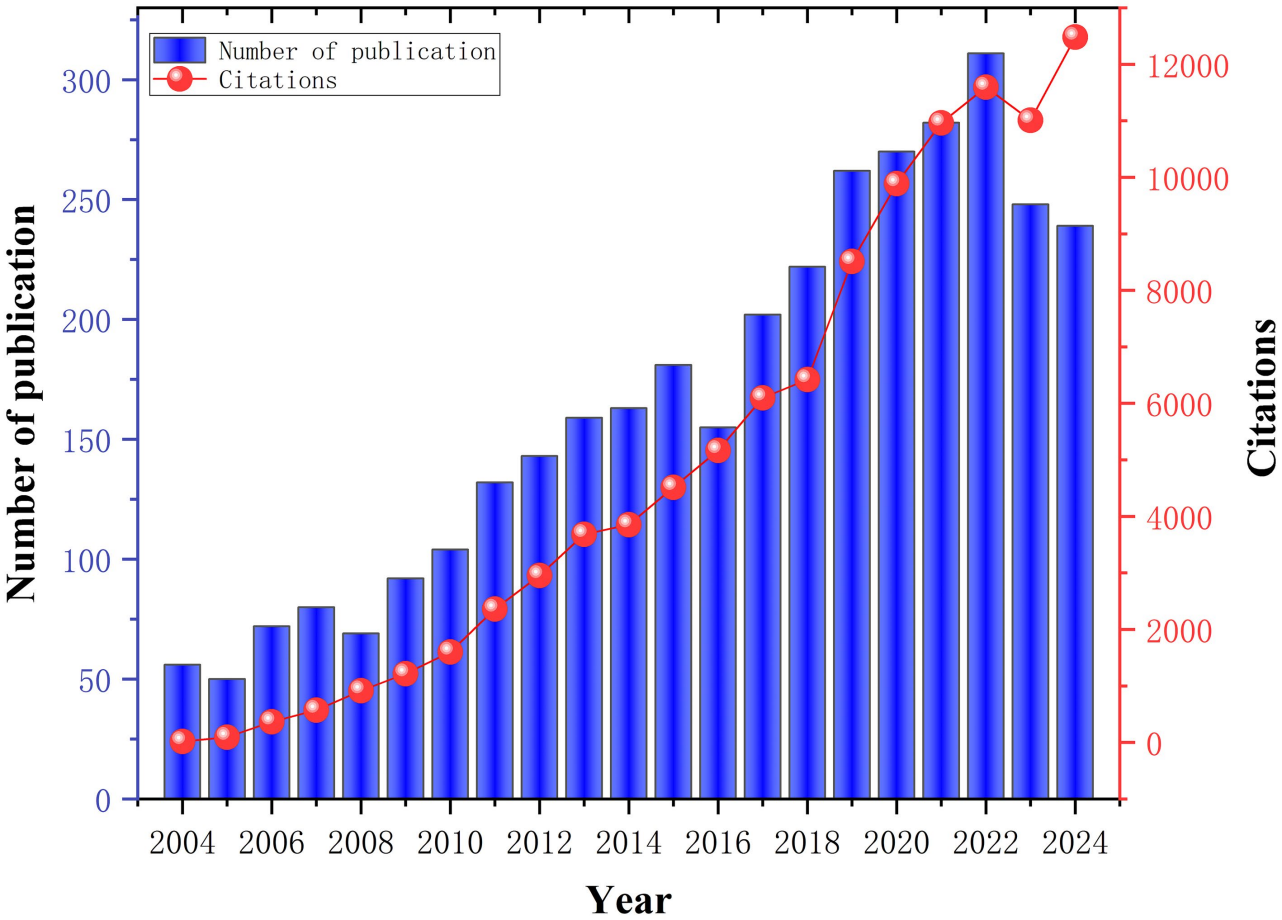
Trends in annual publications and cited articles on post-stroke aphasia between 2004 and 2024.

This study analyzed 3,492 publications contributed by 12,461 authors from 3,569 institutions across 87 countries, published in 533 journals and citing 79,158 articles from 16,530 source journals. As illustrated in Figure 2, PSA research demonstrates a transition from gradual growth to accelerated development. Between 2004 and 2010, the annual output in this field remained limited (56–104 publications/year) with correspondingly few citations, reflecting its nascent stage. Post-2010, driven by advancements in neuroimaging and machine learning, the number of publications increased significantly. By 2015, the annual publication volume exceeded 180 papers, and concurrently, citation counts grew to 4,513. Citations reached 9,890 in 2020, indicating increased global academic and clinical engagement. Publication volume peaked in 2022 with 311 papers. Although a slight downturn was observed in 2023–2024, citation counts remained high at 12,480, suggesting a sustained enhancement of research quality and impact. Despite a transient dip in citations in 2023, the robust recovery in 2024 data suggests that PSA research continues to be in an active phase of technological iteration. This observed growth trajectory aligns with synergistic driving factors, including evolving research paradigms, policy support, funding influx, and disruptive technological innovations. Further deepening of interdisciplinary collaboration is expected to enhance both research depth and translational impact, positioning PSA as a priority area within neurorehabilitation science.

### Analysis of countries/regions and institutions

The 3,492 publications involved contributions from 3,569 institutions across 87 countries/regions. As detailed in Table 1, the top 10 productive countries were ranked by publication volume. The United States emerged as the most active contributor, producing 1,124 publications with the highest citation count (41,373), reflecting its leadership in PSA research. Although its average citation rate (36.81) was surpassed by several other leading nations, its total scholarly output and citation dominance remained unparalleled. Furthermore, its top-ranking Total link strength (TLS) of 616 underscores its central role and extensive collaborative network in the field. The United Kingdom ranked second with 584 publications and 27,032 citations, achieving a high average citation rate (46.29) and the second-highest TLS (600), indicative of both superior research impact and strong international partnerships. China secured third place in productivity with 312 publications but exhibited lower citation metrics (4,272 total citations; 13.69 average) and a comparatively lower TLS (99), likely reflecting its rapid growth phase with a focus potentially more on domestic output or developing international collaborative strength. Germany ranked fourth in productivity (296 publications) but third in citations (15,409) with a notably high average citation rate (52.06) and a significant TLS (302), demonstrating sustained academic influence and strong collaborative ties. Australia, Italy and Canada also demonstrated notable contributions and significant collaborative strengths, particularly Canada with the highest average citation rate among the top 10.

**Table 1:** Publications and citations in the top 10 most productive countries/regions and institutions.

Rank	Country	Publications	Citations	Average citations	Institutions	Publications	Citations	Average citations
1	USA	1,124	41,373	36.81	The University of Queensland	159	4,293	27.00
2	England	584	27,032	46.29	Johne Hopkins University	137	3,843	28.05
3	Australia	365	10,275	28.15	University of Manchester	131	9,034	68.96
4	China	312	4,272	13.69	University College London	96	3,736	38.92
5	Germany	296	15,409	52.06	La Trobe University	95	3,656	38.48
6	Italy	232	10,915	47.05	Boston University	80	2,628	32.85
7	Canada	205	8,730	42.59	University of South Carolina	72	1766	24.53
8	Japan	126	1,683	13.36	Northwest University	69	2,155	31.23
9	Netherlands	125	7,937	63.50	University of Toronto	66	2,475	37.50
10	France	120	7,554	62.95	Edith Cowan University	62	1,515	24.44

**Figure 3:**
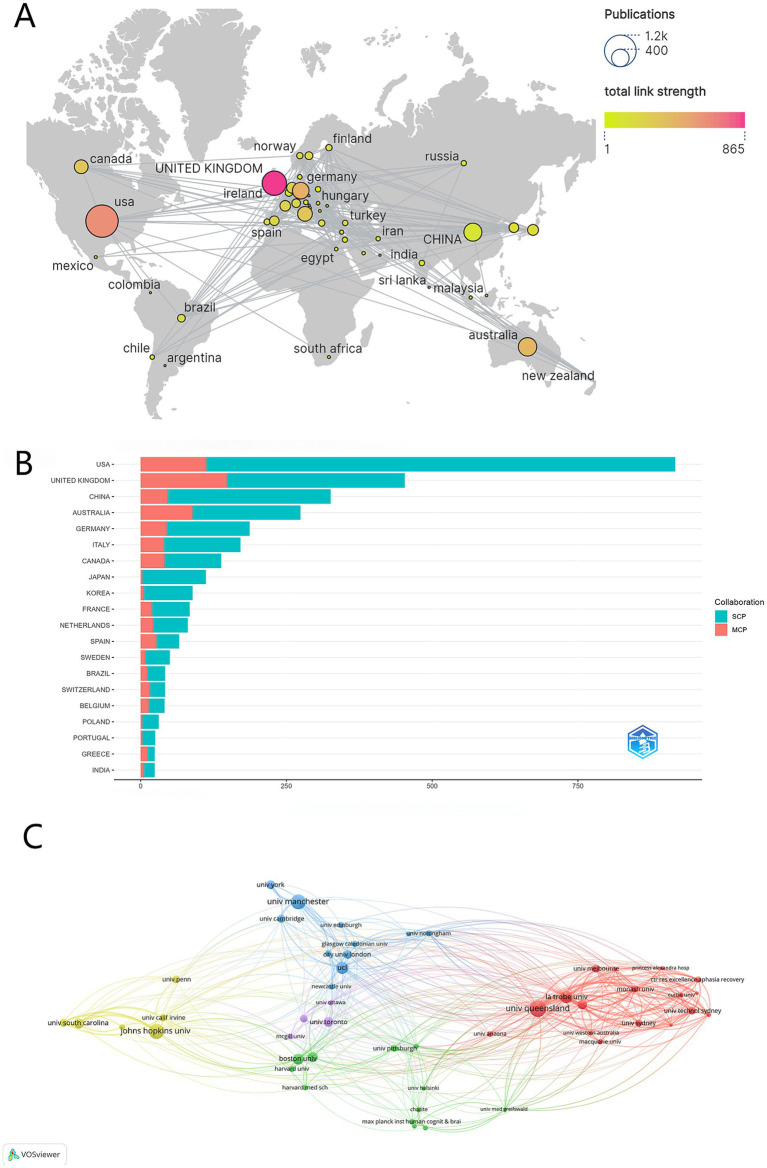
Country/region analysis of the field of post-stroke aphasia. **(A)** Geographic visualization of country/area collaboration. SCP, Single Country Publications; MCP, Multiple Country Publications. **(B)** The top 10 most productive countries/regions. **(C)** Co-analysis of the top 50 most productive institutions in the network visualization map.

Figures 3A,B and Table 1 identified the United States (TLS 616), the United Kingdom (TLS 600), Australia (TLS 346), and Germany (TLS 302) as having the strongest collaborative networks, evidenced by their high Total link strength values, positioning them as central nodes within global research interactions and demonstrating extensive transnational partnerships. Internationally collaborative publications constituted a substantial proportion of influential studies, revealing the indispensable role of global knowledge exchange in driving scientific progress. While China maintains high productivity, its relatively lower TLS (99) compared to other leading nations suggests that while domestic research is strong, its integration strength within multinational consortia might be less pronounced, indicating opportunities for expanded international engagement and network centrality. Bibliometric trends consistently demonstrate that collaborative international research outputs, often originating from countries with high TLS, attract disproportionately greater scholarly attention compared to nationally confined studies, highlighting the strategic advantages of cross-border alliances in amplifying research impact and implementation potential.

Figure 3C visualizes the collaboration network among prominent universities and research institutions involved in this field. The network map utilizes distinct colors to represent institutions from different geographical regions: green nodes denote institutions in the United States, red nodes represent Australian institutions, and blue nodes signify institutions based in the United Kingdom. Institutional analysis reveals the University of Queensland leads significantly in both publications (159) and network centrality (TLS = 448). Key players include Johns Hopkins University (137 publications, TLS = 197) and UK institutions like the University of Manchester (131 publications, TLS = 170) and University College London (96 publications, TLS = 181). A highly collaborative Australian cluster is evident, featuring La Trobe University (TLS = 369) and Edith Cowan University (TLS = 266) alongside UQ. While the US has four top-10 institutions contributing substantial volume, their individual TLS scores are comparatively lower than the top Australian/UK nodes. The University of Toronto is also a notable contributor. Overall, Australian, UK, and US institutions dominate, with TLS pinpointing key collaborative hubs such as UQ and La Trobe.

### Author and co-cited author analysis

**Figure 4:**
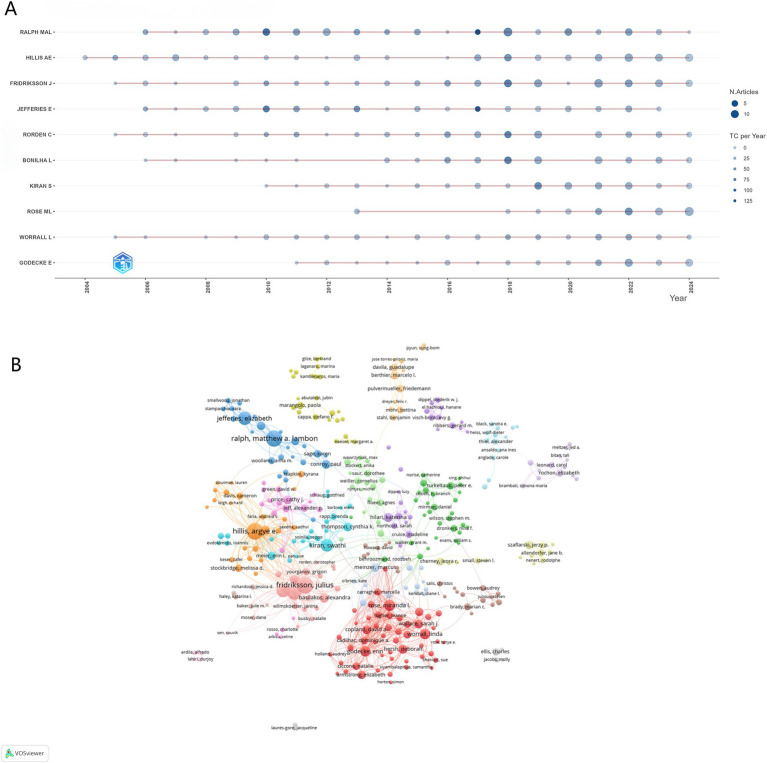
Authors’ contributions to articles on post-stroke aphasia. **(A)** Timeline maps of the number of articles and citations for the top 10 most productive authors. **(B)** Visualization of the top 100 most cited co-cited authors network.

The study identified 12,461 contributing authors in PSA research, with Table 2 and Figure 4A highlighting the top 10 prolific contributors. Fridriksson, j emerged as the most productive author, publishing 91 papers over two decades, followed by Hillis, ae (88 papers) and Ralph, ma (87 papers). Citation metrics reflect scholarly recognition, with 8 of the top 10 authors exceeding 1,000 citations. The H-index, integrating productivity and impact, further validated these authors’ substantial contributions (22). Co-citation analysis revealed Fridriksson, j as the most frequently co-cited author (1,070 citations), indicating foundational influence on PSA research paradigms. Figure 4B illustrates a tightly interconnected co-authorship network among the top 100 cited authors, particularly clustering around high-output researchers. These findings demonstrate sustained scholarly engagement and growing intellectual influence in PSA research, with collaborative networks driving knowledge dissemination and methodological convergence over the 21-year period.

**Table 2:** Top 10 authors and co-cited authors associated with post-stroke aphasia.

Rank	Author	Documents	Citations	Average citations	H-index	Total link strength	Co-cited author	Citations
1	Fridriksson, j	91	4,063	44.65	37	367	Fridriksson, j	1,070
2	Hillis, ae	88	2,466	28.02	31	296	Hillis, ae	863
3	Ralph, ma.	87	7,133	81.99	43	147	Hilari, k	685
4	Bonilha, l	60	2,171	36.18	28	286	Naeser, ma	683
5	Rorden, c	59	3,238	54.88	33	267	Meinzer, m	652
6	Jefferies, e	57	5,152	90.39	29	100	Kertesz, a	634
7	Kiran, s	52	1,237	23.79	21	89	Saur, d	604
8	Rose, ml	41	632	15.41	17	196	Berthier, ml	594
9	Worrall, l	37	1,490	40.27	26	109	Pedersen, pm	560
10	Godecke, e	36	758	21.06	17	207	Jefferies, e	545

### Journal and cited journal visualization analysis

**Figure 5:**
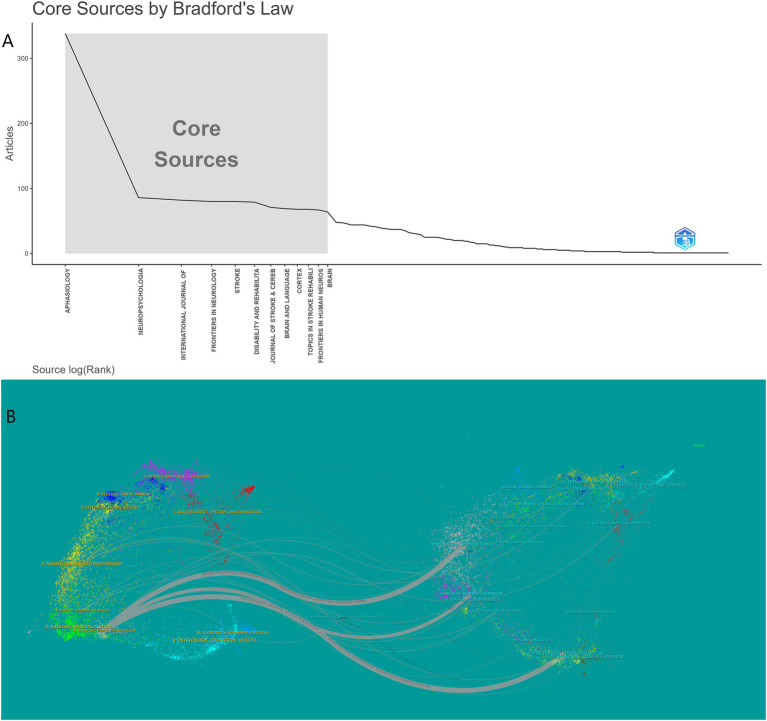
**(A)** Core sources by Bradford’s law. **(B)** Dual map coverage of journals of post-stroke aphasia.

From 2004 to 2024, 533 journals published articles related to PSA. Bradford’s law can identify core journals in a certain field (23) and identified 12 core journals (Figure 5A). Table 3 reveals the multi-dimensional impact landscape of core journals within the PSA research field. In terms of productivity, seven of the top 10 journals belong to the JCR Q1 category, reflecting the field’s strong integration with high-quality clinical neuroscience publications. Among these, Aphasiology ranks first with 350 publications, 6,780 total citations, and a TLS of 3,654. Its TLS value is significantly higher than that of the second-ranked journal, Neuropsychologia (TLS 1,120), indicating that Aphasiology serves not only as a core vehicle for knowledge production but also as a critical hub for cross-institutional and interdisciplinary collaboration. Citation analysis highlights Stroke’s prominence, receiving 7,037 citations and possessing a TLS of 1,921, confirming its pivotal role in foundational stroke research. Notably, despite having only 81 publications, Disability and Rehabilitation garnered 1,907 citations and achieved a TLS of 1,174, revealing the high penetration rate of its research findings within the rehabilitation medicine network. The divergence observed between TLS and Impact Factor merits attention. For instance, the International Journal of Language & Communication Disorders (IF = 1.5) possesses a TLS of 754, surpassing that of some Q1 journals. This suggests that traditional IF metrics may underestimate the structural importance of specialized language pathology journals within the PSA collaborative network.

**Table 3:** The top 10 published journals and the top 10 co-cited journals in the field of post-stroke aphasia.

Rank	Journal	Documents	Citations	Total link strength	JCR	Impact factor (2024)	Co-cited journal	Citations	JCR	Impact factor (2024)
1	Aphasiology	350	6,780	3,654	2	1.5	Aphasiology	9,500	2	1.5
2	Neuropsychologia	86	2,728	1,120	3	2	Stroke	8,791	1	7.8
3	International journal of language & communication disorders	82	1,122	754	1	1.5	Brain	6,887	1	10.6
4	Disability and rehabilitation	81	1907	1,174	1	2.1	Brain and language	5,740	1	2.1
5	Frontiers in neurology	80	1,147	986	2	2.7	Neuroimage	5,606	1	4.7
6	Stroke	80	7,037	1921	1	7.8	Neurology	4,185	1	7.7
7	Journal of stroke & cerebrovascular diseases	74	1,008	304	1	2	Neuropsychologia	3,680	3	2
8	Brain and language	69	3,453	1774	1	2.1	Cortex	2,779	1	3.2
9	Cortex	69	2,586	1,290	1	3.2	Archives of physical medicine and rehabilitation	2,475	1	3.6
10	Topics in stroke rehabilitation	69	1,393	677	1	2.2	Annals of neurology	2,256	1	8.1

The dual-map overlay analysis (Figure 5B) delineates the interdisciplinary citation relationships and knowledge flow within PSA research (24). On the left, citing disciplines are primarily concentrated in areas encompassing Medicine, Medical, and Clinical sciences; Psychology, Education, and Social sciences; and Neurology, Sports, and Ophthalmology, representing the principal origins of the analyzed publications. On the right, key cited disciplinary areas include Molecular, Biology, and Genetics; Health, Nursing, and Medicine; and Psychology, Education, and Health. Significant citation trajectories reveal the primary knowledge flows: pathways originating from the Medicine, Medical, and Clinical discipline heavily target work within both the Health, Nursing, and Medicine area and the Psychology, Education, and Health area. Furthermore, a strong connection exists from the Psychology, Education, and Social sciences discipline to the Psychology, Education, and Health area. These pathways demonstrate that research originating from clinical medicine and psychology domains builds substantially upon foundational work within the health, nursing, and broader psychological and educational sciences. This visualization underscores the field’s inherent interdisciplinarity, illustrating the integration of clinical and psychological knowledge with health services, nursing, and psychological science research, crucial for advancing cognitive rehabilitation, understanding neural mechanisms, and developing effective interventions.

### Analysis of co-cited references

Co-citation analysis identifies foundational works frequently referenced together in subsequent studies. Table 4 lists the top 10 co-cited references, predominantly clinical trials and systematic reviews. The seminal study “Dynamics of language reorganization after stroke” (25) delineated a triphasic neural reorganization framework through longitudinal fMRI investigations. Initial observations revealed diminished activation within left-hemisphere language regions during the acute phase, with residual inferior frontal gyrus activity demonstrating direct correlation with preserved baseline linguistic function. Subsequent neuroplastic adaptations featured bilateral cortical recruitment patterns, particularly involving right-hemisphere homologs of Broca’s area and supplementary motor regions, a mechanism strongly predictive of functional recovery outcomes. The chronic phase manifested progressive restoration of left-lateralized activation architecture concurrent with sustained clinical improvement, completing the neural reorganization continuum. This temporal trajectory underscores the interdependence between early right-hemisphere compensatory mechanisms and gradual left-hemisphere functional restitution during post-stroke language recovery.

**Table 4:** The research on post-stroke aphasia was cited as the top 10 literature.

Rank	Co-cited reference	Citations	Total link strength	Journal	Types
1	Dynamics of language reorganization after stroke. Brain. 2006 Jun;129(Pt 6):1371–84. doi: 10.1093/brain/awl090. Epub 2006 Apr 25. PMID: 16638796.	344	10,751	Brain	Article
2	Western Aphasia Battery,1982	306	5,536	none	book
3	Aphasia in acute stroke: incidence, determinants, and recovery. Ann Neurol. 1995 Oct;38(4):659–66. doi: 10.1002/ana.410380416. PMID: 7574464.	279	6,074	Annals of neurology	Article
4	Epidemiology of aphasia attributable to first ischemic stroke: incidence, severity, fluency, etiology, and thrombolysis. Stroke. 2006 Jun;37(6):1379–84. doi: 10.1161/01.STR.0000221815.64093.8c. Epub 2006 May 11. PMID: 16690899.	270	5,176	Stroke	Article
5	Speech and language therapy for aphasia following stroke. Cochrane Database Syst Rev. 2016 Jun 1;2016(6):CD000425. doi: 10.1002/14651858.CD000425.pub4. PMID: 27245310; PMCID: PMC8078645.	239	5,129	Cochrane Database Syst Rev	review
6	Aphasia in acute stroke and relation to outcome. J Intern Med. 2001 May;249(5):413–22. doi: 10.1046/j.1365-2796.2001.00812.x. PMID: 11350565.	231	5,053	Journal of Internal Medicine	Article
7	The cortical organization of speech processing. Nat Rev. Neurosci. 2007 May;8(5):393–402. doi: 10.1038/nrn2113. Epub 2007 Apr 13. PMID: 17431404.	225	6,481	Nature reviews neuroscience	Review
8	Aphasia after stroke: type, severity and prognosis. The Copenhagen aphasia study. Cerebrovasc Dis. 2004;17(1):35–43. doi: 10.1159/000073896. Epub 2003 Oct 3. PMID: 14530636.	222	5,341	Cerebrovascular Diseases	Article
9	Improved picture naming in chronic aphasia after TMS to part of right Broca’s area: an open-protocol study. Brain Lang. 2005 Apr;93(1):95–105. doi: 10.1016/j.bandl.2004.08.004. PMID: 15766771.	205	6,822	Brain Lang	Clinical trial
10	Intensity of aphasia therapy, impact on recovery. Stroke. 2003 Apr;34(4):987–93. doi: 10.1161/01.STR.0000062343.64383.D0. Epub 2003 Mar 20. PMID: 12649521.	198	4,852	Stroke	Article

**Figure 6:**
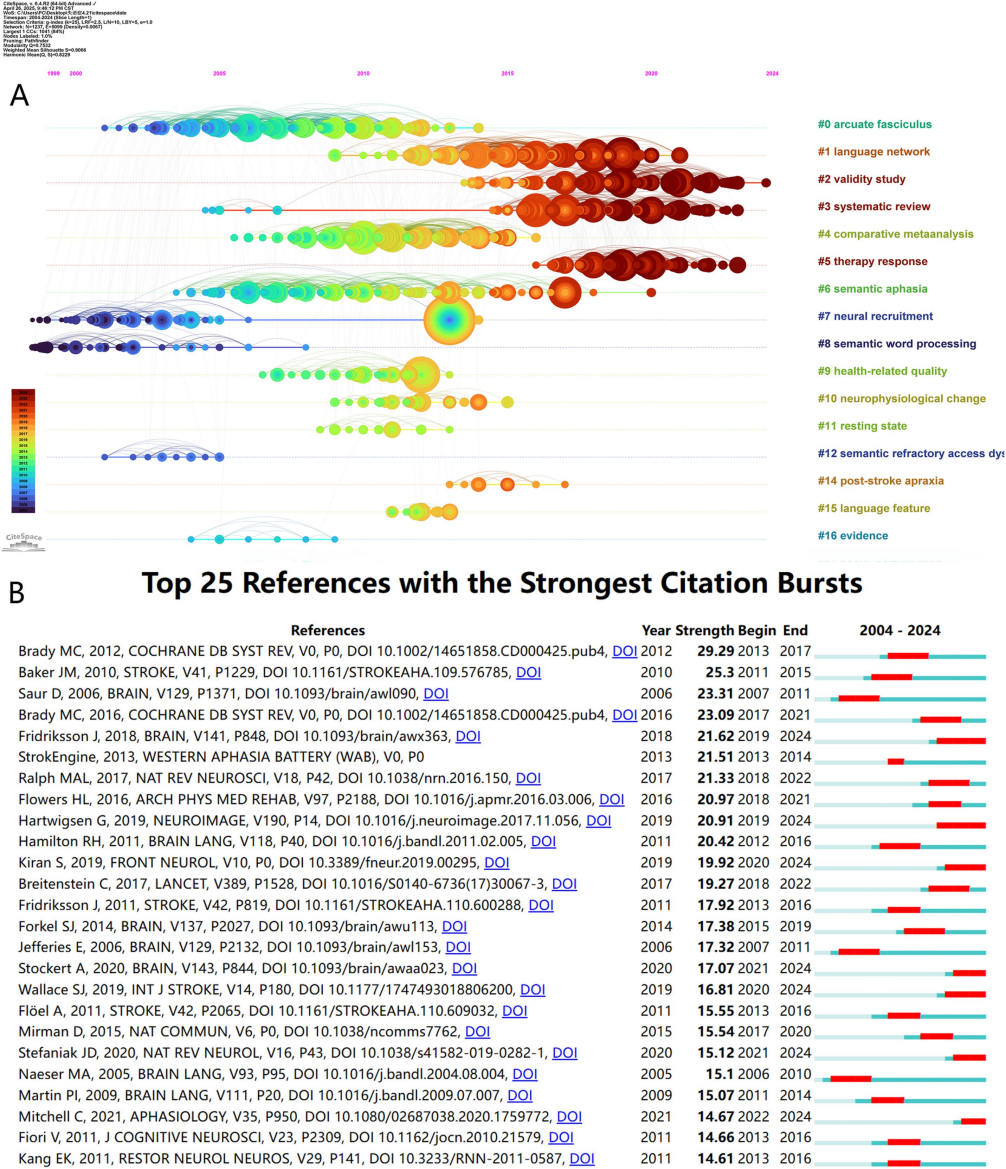
**(A)** The timeline view of the co-cited references network. **(B)** The top 25 references with the strongest citation bursts.

Figure 6A presents a timeline visualization of co-cited reference clusters. Nodes positioned along the same horizontal line constitute a cluster, the theme of which is indicated by the label presented on the right. Node size is proportional to co-citation frequency. The timeline progresses from left to right, with earlier nodes appearing on the left and later nodes on the right. As depicted, several recent research hotspots identified include ‘language network’, ‘validity study’, ‘systematic review’, ‘therapy response’, and ‘semantic aphasia’.

Burst analysis of co-cited references (Figure 6B) allows for the investigation of the persistence of research hotspots in the PSA field. Figure 6B visually represents this analysis. The timeline spans 2004–2024, and the red bars indicate the period during which a reference experienced a citation burst. Notably, the article ‘Speech and language therapy for aphasia following stroke’ authored by Brady MC in 2012 exhibited the highest burst strength (29.29), active between 2013 and 2017. This pivotal work assessed the efficacy of speech and language therapy (SLT) for post-stroke aphasia, demonstrating significant benefits over no treatment in improving functional communication, reading, and expressive language, although long-term effects remained unclear; it also suggested higher intensity SLT might be more effective but potentially associated with higher dropout rates, highlighting the need for more high-quality comparative intervention studies (26). Additionally, other references exhibiting significant citation bursts include Baker JM’s work ‘Using transcranial direct-current stimulation to treat stroke patients with aphasia’ (burst 2011–2015, strength 25.3) and Saur D’s study ‘Dynamics of language reorganization after stroke’ (burst 2007–2011, strength 23.31).

### Analysis of keywords

**Figure 7:**
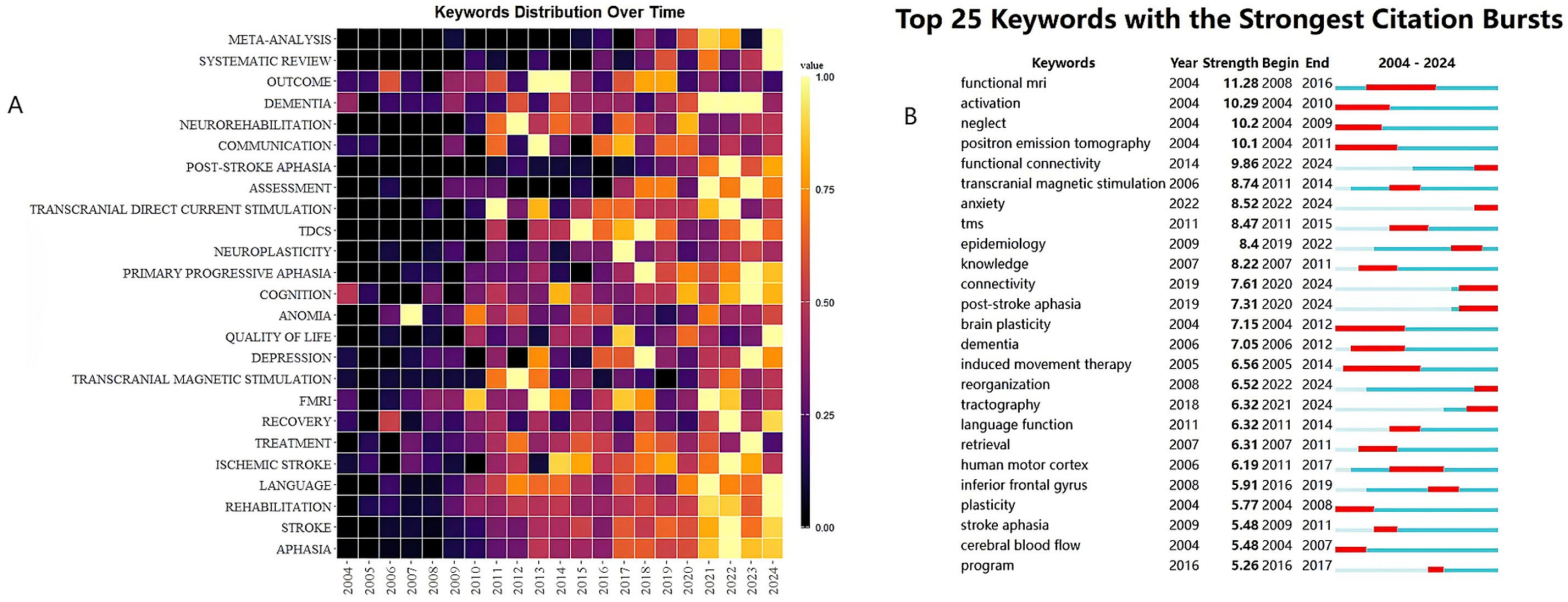
**(A)** The yearly occurrences of top 25 keywords. **(B)** Top 25 keywords with the strongest citation bursts.

Figure 7A systematically visualizes the 21-year disciplinary evolution of post-stroke language rehabilitation research through keyword frequency trends displayed as a heatmap. Foundational clinical concepts, notably ‘STROKE’ and ‘APHASIA’, persist as high-frequency nodes across the timeline, establishing the field’s theoretical basis. A significant paradigm shift occurred post-2010, marked by the rising prominence of neuroimaging technologies such as ‘FMRI’, which shifted focus from symptom observation towards brain network visualization. Therapeutic innovations reveal a generational progression, moving from earlier conventional ‘LANGUAGE’-centric approaches towards the rapid emergence and sustained prevalence of non-invasive neuromodulation techniques, including ‘TRANSCRANIAL DIRECT CURRENT STIMULATION’ (tDCS), after 2015. This trend coincides with an increased emphasis on ‘NEUROPLASTICITY’ and ‘COGNITION’, indicating intensified mechanistic investigations. Temporal asymmetry in intervention priorities is also apparent; acute-phase terms like ‘THROMBOLYSIS’ showed peak frequencies around 2010–2016, while chronic-phase concepts, including ‘DEPRESSION’ and ‘QUALITY OF LIFE’, gained frequency after 2020, reflecting a move towards ‘whole-cycle rehabilitation.’ Current research differentiates across technical dimensions, exemplified by the focus on neuromodulation with terms like ‘tDCS’; clinical dimensions, involving keywords such as ‘TREATMENT’ and ‘NEUROREHABILITATION’; and social dimensions, highlighted by the growing frequency of ‘COMMUNICATION’. This multidimensional evolution underscores the need for a comprehensive ‘biopsychosocial’ transmodal framework that integrates physiological, cognitive-emotional, and community engagement strategies to optimize patient recovery.

Figure 7B, generated through keyword burst-intensity analysis spanning 2004 to 2024, reveals three distinct evolutionary stages in post-stroke language rehabilitation research. The initial research period (2004–2012) was dominated by PET and fMRI technologies, primarily targeting lesion-symptom mapping to localize language deficit correlates. Subsequent advancements (2013–2018) transitioned toward transcranial magnetic stimulation (TMS) applications and white matter remodeling studies, deepening mechanistic insights into neuroplasticity-driven recovery pathways. The current phase has been characterized by the integration of functional connectomics and epidemiological datasets, expanding into precision therapeutic personalization and population-level burden assessments. This trajectory highlights a progressive shift from anatomical correlation studies to dynamic network-based interventions, while underscoring persistent gaps in translating neuroimaging discoveries into clinically actionable protocols.

A persistent translational gap exists between neuroimaging advancements and clinical translation, with the majority of mechanistic discoveries from advanced imaging modalities remaining confined to experimental research rather than informing therapeutic strategies. Addressing this challenge requires integrating multimodal neuromodulation approaches, comprehensive rehabilitation frameworks spanning acute to chronic recovery phases, and intelligent digital therapeutics. This multidimensional synthesis will catalyze dual-axis innovation—enhancing neurobiological circuit reorganization through targeted interventions while improving functional communication outcomes for community reintegration—effectively closing the divide between laboratory insights and clinically meaningful functional restoration.

## Discussion

This study elucidates the paradigm evolution in post-stroke aphasia mechanism research, characterized by three distinct developmental phases. Early investigations focused on anatomical localization of language deficits through PET and structural MRI, aligning with the critical zone lesion hypothesis proposed by Gorno-Tempini (27). The subsequent phase shifted toward white matter integrity evaluation using DTI and resting-state fMRI, culminating in the landmark triphasic dynamic reorganization model of language networks by Hartwigsen (28). Current research has advanced into network-level analyses through functional connectomics and machine learning, exemplified by Yourganov’s (29) graph-theoretical exploration of compensatory interactions between default mode and language networks. This trajectory reflects neuroscience’s overarching transition from structural localization to systemic functional integration.

Despite the dominance of neuroimaging modalities in mechanistic exploration, their clinical translation efficiency remains suboptimal. Our co-occurrence network analysis reveals limited correlational studies between imaging biomarkers and clinical outcome measures, predominantly confined to cross-sectional research designs. This observation aligns with the “neuroimaging-behavioral decoupling paradox” articulated by Marek (30), wherein micro-scale neural remodeling fails to directly account for macro-scale functional recovery variability. Notably, while non-invasive neuromodulation technologies experienced exponential growth post-2015, clinical trials demonstrate their effect sizes fall significantly below preclinical animal models, indicating suboptimal parameter translation (31). This gap necessitates establishing a closed-loop framework integrating computational modeling guidance (32), personalized neuromodulation targeting, and multimodal outcome evaluation to bridge the precision medicine divide between mechanistic insights and therapeutic optimization (33).

The international collaboration landscape in post-stroke aphasia research reveals a pronounced core-periphery hierarchy, with the United States, Australia, and the United Kingdom serving as central hubs that steer research priorities and methodological frameworks. These core nations exert disproportionate influence over trial design and theoretical development, particularly evident in neuromodulation research where their institutional paradigms dominate global clinical protocols. China, despite ranking fourth in publication volume, maintains limited integration within international networks, reflecting systemic imbalances in collaborative knowledge production. This structural disparity perpetuates the marginalization of region-specific clinical features, as evidenced by the insufficient integration of East Asian linguistic profiles and aphasia subtype patterns into dominant neurocognitive models. To counterbalance this asymmetry, strategic prioritization of multinational registry studies is imperative, fostering equitable data representation across diverse populations while strengthening cross-cultural validation of therapeutic frameworks. Such initiatives would enhance the ecological validity of neurorehabilitation strategies while addressing critical gaps in global health equity (34).

Keyword chronology analysis reveals a pronounced acute-phase bias in current research, with hyperacute intervention studies predominating while chronic-phase management investigations remain underrepresented. This imbalance contrasts sharply with clinical realities, as epidemiological evidence indicates a substantial proportion of PSA patients experience persistent symptoms into chronic stages (35). Although recent studies have begun addressing long-term neuroplasticity, cross-stage longitudinal investigations remain scarce (36, 37). Dual-map overlay analysis demonstrates accelerating convergence among psychology, engineering, and clinical neurology, exemplified by emerging applications such as virtual reality-enabled contextualized language training (38) and natural language processing (NLP)-driven automated aphasia severity assessment (39). However, these technological advancements remain largely confined to proof-of-concept development stages, confronting systemic implementation barriers across three interconnected dimensions. Technical limitations emerge from inadequate generalizability of algorithmic models across diverse patient demographics and linguistic symptom profiles (40, 41). Concurrently, unresolved ethical considerations surrounding artificial intelligence applications, particularly transparency deficits in decision-making architectures and data governance frameworks, hinder clinical adoption (42, 43). These challenges become compounded by infrastructural disparities in digital healthcare readiness across global healthcare systems, creating implementation inequities that demand coordinated interdisciplinary efforts spanning computational neuroscience, clinical ethics, and health policy domains.

## Limitations

Several limitations of this study should be acknowledged. First, the analysis was restricted to data from the Web of Science Core Collection (WoSCC) to ensure software compatibility, potentially excluding relevant studies indexed in other databases such as PubMed, Google Scholar, and Embase. Second, emerging high-quality publications may not have received sufficient representation due to lower citation frequencies characteristic of recently published works. Finally, the exclusive inclusion of English-language literature may introduce selection bias, particularly regarding region-specific research contributions. These constraints should inform cautious interpretation of the findings while highlighting opportunities for methodological refinement in future bibliometric investigations.

## Conclusion

Bibliometric analysis reveals three paradigm shifts in post-stroke aphasia research: from lesion localization to neural reorganization mechanisms, and ultimately to dynamic brain network investigations. Despite technological progress, significant translational gaps persist between laboratory discoveries and clinical practice. Advanced neuroimaging techniques seldom integrate functional assessment tools, while neuromodulation approaches demonstrating preclinical efficacy show reduced effectiveness in human applications. International collaboration patterns exhibit a core-periphery structure dominated by developed nations, with emerging contributors like China requiring enhanced development of localized assessment instruments and deeper multinational cooperation. Current research disproportionately focuses on acute-phase interventions, with digital therapeutics and chronic-phase management strategies remaining underdeveloped. Future priorities should emphasize personalized neuromodulation protocols, comprehensive rehabilitation frameworks spanning the full recovery cycle, and intelligent therapeutic systems. Concurrently, accelerating the establishment of multinational neuroimaging databases and culturally adaptive assessment instruments will bridge the research-clinical translation gap. This investigation provides visualized insights into post-stroke aphasia research landscapes, offering researchers a multidimensional perspective to advance understanding and innovation in the field.

## Data Availability

The original contributions presented in the study are included in the article/Supplementary material, further inquiries can be directed to the corresponding author.

## References

[1] Engelter ST Gostynski M Papa S Frei M Born C Ajdacic-Gross V . Epidemiology of aphasia attributable to first ischemic stroke: incidence, severity, fluency, etiology, and thrombolysis. Stroke. (2006) 37:1379–84. doi: 10.1161/01.STR.0000221815.64093.8c, PMID: 16690899

[2] Khedr EM Abbass MA Soliman RK Zaki AF Gamea A El-Fetoh NA . A hospital-based study of post-stroke aphasia: frequency, risk factors, and topographic representation. Egyptian J Neurol, Psychiatry Neurosurg. (2019) 56:2. doi: 10.1186/s41983-019-0128-1, PMID: 40335205

[3] Tak HJ Jang SH. Relation between aphasia and arcuate fasciculus in chronic stroke patients. BMC Neurol. (2014) 14:46. doi: 10.1186/1471-2377-14-46, PMID: 24607148 PMC3973830

[4] Gajardo-Vidal A Lorca-Puls DL Warner H Pshdary B Crinion JT . Damage to Broca's area does not contribute to long-term speech production outcome after stroke. Brain. (2021) 144:817–32. doi: 10.1093/brain/awaa460, PMID: 33517378 PMC8041045

[5] Jang SH. Diffusion tensor imaging studies on arcuate fasciculus in stroke patients: a review. Front Hum Neurosci. (2013) 7:749. doi: 10.3389/fnhum.2013.00749 24198780 PMC3814569

[6] Hillis AE Newhart M Heidler J Barker P Herskovits E Degaonkar M. The roles of the "visual word form area" in reading. NeuroImage. (2005) 24:548–59. doi: 10.1016/j.neuroimage.2004.08.026, PMID: 15627597

[7] Brauer J Anwander A Perani D Friederici AD. Dorsal and ventral pathways in language development. Brain Lang. (2013) 127:289–95. doi: 10.1016/j.bandl.2013.03.001, PMID: 23643035

[8] Fridriksson J den Ouden DB Hillis AE Hickok G Rorden C Basilakos A . Anatomy of aphasia revisited. Brain. (2018) 141:848–62. doi: 10.1093/brain/awx363, PMID: 29360947 PMC5837461

[9] Hartwigsen G Volz LJ. Probing rapid network reorganization of motor and language functions via neuromodulation and neuroimaging. NeuroImage. (2021) 224:117449. doi: 10.1016/j.neuroimage.2020.117449, PMID: 33059054

[10] Hamilton RH Chrysikou EG Coslett B. Mechanisms of aphasia recovery after stroke and the role of noninvasive brain stimulation. Brain Lang. (2011) 118:40–50. doi: 10.1016/j.bandl.2011.02.005, PMID: 21459427 PMC3109088

[11] Jiang Z Kuhnke P Stockert A Wawrzyniak M Halai A Saur D . Dynamic reorganization of task-related network interactions in post-stroke aphasia recovery. Brain. (2025). doi: 10.1093/brain/awaf036, PMID: 39883566 PMC12493039

[12] Finc K Bonna K He X Lydon-Staley DM Kühn S Duch W . Dynamic reconfiguration of functional brain networks during working memory training. Nat Commun. (2020) 11:2435. doi: 10.1038/s41467-020-15631-z, PMID: 32415206 PMC7229188

[13] Liljestrom M Kujala J Stevenson C Salmelin R. Dynamic reconfiguration of the language network preceding onset of speech in picture naming. Hum Brain Mapp. (2015) 36:1202–16. doi: 10.1002/hbm.22697, PMID: 25413681 PMC4365727

[14] Li B Deng S Zhuo B Sang B Chen J Zhang M . Effect of acupuncture vs sham acupuncture on patients with Poststroke motor aphasia: a randomized clinical trial. JAMA Netw Open. (2024) 7:e2352580. doi: 10.1001/jamanetworkopen.2023.52580, PMID: 38252438 PMC10804271

[15] Ninkov A Frank JR Maggio LA. Bibliometrics: methods for studying academic publishing. Perspect Med Educ. (2022) 11:173–6. doi: 10.1007/s40037-021-00695-4, PMID: 34914027 PMC9240160

[16] Thompson DF Walker CK. A descriptive and historical review of bibliometrics with applications to medical sciences. Pharmacotherapy. (2015) 35:551–9. doi: 10.1002/phar.1586, PMID: 25940769

[17] Chen C. Searching for intellectual turning points: progressive knowledge domain visualization. Proc Natl Acad Sci USA. (2004) 101:5303–10. doi: 10.1073/pnas.0307513100 14724295 PMC387312

[18] Synnestvedt MB Chen C Holmes JH. CiteSpace II: visualization and knowledge discovery in bibliographic databases. AMIA Annu Symp Proc. (2005) 2005:724–8. 16779135 PMC1560567

[19] van Eck NJ Waltman L. Software survey: VOSviewer, a computer program for bibliometric mapping. Scientometrics. (2010) 84:523–38. doi: 10.1007/s11192-009-0146-3, PMID: 20585380 PMC2883932

[20] Page MJ McKenzie JE Bossuyt PM Boutron I Hoffmann TC Mulrow CD . The PRISMA 2020 statement: an updated guideline for reporting systematic reviews. BMJ. (2021) 372:n71. doi: 10.1136/bmj.n71, PMID: 33782057 PMC8005924

[21] Aria M Cuccurullo C. Bibliometrix: an R-tool for comprehensive science mapping analysis. J Inf Secur. (2017) 11:959–75. doi: 10.1016/j.joi.2017.08.007

[22] Roldan-Valadez E Salazar-Ruiz SY Ibarra-Contreras R Rios C. Current concepts on bibliometrics: a brief review about impact factor, Eigenfactor score, CiteScore, SCImago journal rank, source-normalised impact per paper, H-index, and alternative metrics. Ir J Med Sci. (2019) 188:939–51. doi: 10.1007/s11845-018-1936-5, PMID: 30511320

[23] Venable GT Shepherd BA Loftis CM McClatchy SG Roberts ML Fillinger ME . Bradford's law: identification of the core journals for neurosurgery and its subspecialties. J Neurosurg. (2016) 124:569–79. doi: 10.3171/2015.3.JNS15149, PMID: 26339849

[24] Chen C Leydesdorff L. Patterns of connections and movements in dual-map overlays: a new method of publication portfolio analysis. J Assoc Inf Sci Technol. (2013) 65:334–51. doi: 10.1002/asi.22968, PMID: 40349218

[25] Saur D Lange R Baumgaertner A Schraknepper V Willmes K Rijntjes M . Dynamics of language reorganization after stroke. Brain. (2006) 129:1371–84. doi: 10.1093/brain/awl090, PMID: 16638796

[26] Brady MC Kelly H Godwin J Enderby P Campbell P. Speech and language therapy for aphasia following stroke. Cochrane Database Syst Rev. (2016) 2016:CD000425. doi: 10.1002/14651858.CD000425.pub4, PMID: 27245310 PMC8078645

[27] Gorno-Tempini ML Hillis AE Weintraub S Kertesz A Mendez M Cappa SF . Classification of primary progressive aphasia and its variants. Neurology. (2011) 76:1006–14. doi: 10.1212/WNL.0b013e31821103e6, PMID: 21325651 PMC3059138

[28] Hartwigsen G Saur D. Neuroimaging of stroke recovery from aphasia - insights into plasticity of the human language network. NeuroImage. (2019) 190:14–31. doi: 10.1016/j.neuroimage.2017.11.056, PMID: 29175498

[29] Yourganov G Fridriksson J Rorden C Gleichgerrcht E Bonilha L. Multivariate connectome-based symptom mapping in post-stroke patients: networks supporting language and speech. J Neurosci. (2016) 36:6668–79. doi: 10.1523/JNEUROSCI.4396-15.2016, PMID: 27335399 PMC4916245

[30] Marek S Tervo-Clemmens B Calabro FJ Montez DF Kay BP Hatoum AS . Reproducible brain-wide association studies require thousands of individuals. Nature. (2022) 603:654–60. doi: 10.1038/s41586-022-04492-9, PMID: 35296861 PMC8991999

[31] Lefaucheur JP Antal A Ayache SS Benninger DH Brunelin J Cogiamanian F . Evidence-based guidelines on the therapeutic use of transcranial direct current stimulation (tDCS). Clin Neurophysiol. (2017) 128:56–92. doi: 10.1016/j.clinph.2016.10.087, PMID: 27866120

[32] Fedorenko E Ivanova AA Regev TI. The language network as a natural kind within the broader landscape of the human brain. Nat Rev Neurosci. (2024) 25:289–312. doi: 10.1038/s41583-024-00802-4, PMID: 38609551 PMC13222024

[33] Pulvermüller F. Neurobiological mechanisms for language, symbols and concepts: clues from brain-constrained deep neural networks. Prog Neurobiol. (2023) 230:102511. doi: 10.1016/j.pneurobio.2023.102511, PMID: 37482195 PMC10518464

[34] Iorga M Higgins J Caplan D Zinbarg R Kiran S Thompson CK . Predicting language recovery in post-stroke aphasia using behavior and functional MRI. Sci Rep. (2021) 11:8419. doi: 10.1038/s41598-021-88022-z, PMID: 33875733 PMC8055660

[35] Cichon N Wlodarczyk L Saluk-Bijak J Bijak M Redlicka J Gorniak L . Novel advances to post-stroke aphasia pharmacology and rehabilitation. J Clin Med. (2021) 10:3778. doi: 10.3390/jcm10173778, PMID: 34501229 PMC8432240

[36] Varkanitsa M Kiran S. Understanding, facilitating and predicting aphasia recovery after rehabilitation. Int J Speech Lang Pathol. (2022) 24:248–59. doi: 10.1080/17549507.2022.2075036, PMID: 35603543 PMC9398975

[37] Kiran S Thompson CK. Neuroplasticity of language networks in aphasia: advances, updates, and future challenges. Front Neurol. (2019) 10:295. doi: 10.3389/fneur.2019.00295, PMID: 31001187 PMC6454116

[38] Devane N Behn N Marshall J Ramachandran A Wilson S Hilari K. The use of virtual reality in the rehabilitation of aphasia: a systematic review. Disabil Rehabil. (2023) 45:3803–22. doi: 10.1080/09638288.2022.2138573, PMID: 36326199

[39] Day M Dey RK Baucum M Paek EJ Park H Khojandi A. Predicting severity in people with aphasia: a natural language processing and machine learning approach. Annu Int Conf IEEE Eng Med Biol Soc. (2021) 2021:2299–302. doi: 10.1109/EMBC46164.2021.9630694, PMID: 34891746

[40] Zhong X. AI-assisted assessment and treatment of aphasia: a review. Front Public Health. (2024) 12:1401240. doi: 10.3389/fpubh.2024.1401240, PMID: 39281082 PMC11394183

[41] Privitera AJ Ng SHS Kong AP Weekes BS. AI and aphasia in the digital age: a critical review. Brain Sci. (2024) 14:383. doi: 10.3390/brainsci14040383, PMID: 38672032 PMC11047933

[42] Aravazhi PS Gunasekaran P Benjamin NZY Thai A Chandrasekar KK Kolanu ND . The integration of artificial intelligence into clinical medicine: trends, challenges, and future directions. Dis Mon. (2025) 101882. [epub ahead of print]. doi: 10.1016/j.disamonth.2025.101882 40140300

[43] Olawade DB Weerasinghe K Teke J Msiska M Boussios S Hatzidimitriadou E. Evaluating AI adoption in healthcare: insights from the information governance professionals in the United Kingdom. Int J Med Inform. (2025) 199:105909. doi: 10.1016/j.ijmedinf.2025.105909, PMID: 40198930

