# Supplementary material for: Research trends in post-stroke aphasia (2004–2024): a bibliometric and visualized analysis
Source: Front Neurol. 2025 Jun 25;16:1588130. doi: 10.3389/fneur.2025.1588130 (PMC12238218; doi:10.3389/fneur.2025.1588130)
Supplement: Supplementary file 1 [file Data_Sheet_1.pdf]

post-stroke aphasia
aphasia after stroke

Entry Terms:

- Strokes
- Cerebrovascular Accident
- Cerebrovascular Accidents
- Cerebral Stroke
- Cerebral Strokes
- Stroke, Cerebral
- Strokes, Cerebral
- Cerebrovascular Apoplexy
- Apoplexy, Cerebrovascular
- Vascular Accident, Brain
- Brain Vascular Accident
- Brain Vascular Accidents
- Vascular Accidents, Brain
- Cerebrovascular Stroke
- Cerebrovascular Strokes
- Stroke, Cerebrovascular
- Strokes, Cerebrovascular
- Apoplexy
- CVA (Cerebrovascular Accident)
- CVAs (Cerebrovascular Accident)
- Stroke, Acute

- Acute Stroke
- Acute Strokes
- Strokes, Acute
- Cerebrovascular Accident, Acute
- Acute Cerebrovascular Accident
- Acute Cerebrovascular Accidents
- Cerebrovascular Accidents, Acute

Entry Terms:

- Word Deafness
- Deafness, Word
- Alogia
- Alogias
- Anepia
- Anepias
- Logagnosia
- Logagnosias
- Logamnesia
- Logamnesias
- Logasthenia
- Logasthenias
- Aphasia, Acquired
- Acquired Aphasia
- Dysphasia

Aphasia, Ageusic

Ageusic Aphasia

Ageusic Aphasias

Aphasia, Auditory Discriminatory

Auditory Discriminatory Aphasia

Auditory Discriminatory Aphasias

Discriminatory Aphasia, Auditory

Discriminatory Aphasias, Auditory

Aphasia, Commisural

Aphasias, Commisural

Commisural Aphasia

Commisural Aphasias

Aphasia, Functional

Functional Aphasia

Functional Aphasias

Aphasia, Global

Global Aphasia

Global Aphasias

Dysphasia, Global

Global Dysphasia

Global Dysphasias

Aphasia, Graphomotor

Graphomotor Aphasia

Graphomotor Aphasias

Aphasia, Intellectual

Intellectual Aphasia

Intellectual Aphasias

Aphasia, Mixed

Mixed Aphasia

Mixed Aphasias

Aphasia, Post-Ictal

Aphasia, Post Ictal

Post-Ictal Aphasia

Post-Ictal Aphasias

Aphasia, Post-Traumatic

Aphasia, Post Traumatic

Post-Traumatic Aphasia

Post-Traumatic Aphasias

Aphasia, Progressive

Progressive Aphasia

Progressive Aphasias

Dejerine-Lichtheim Phenomenon

Dejerine Lichtheim Phenomenon

Phenomenon, Dejerine-Lichtheim

Lichtheim's Sign

Lichtheim Sign

Lichtheims Sign

Sign, Lichtheim's

Aphasia, Semantic

Semantic Aphasia

Semantic Aphasias

Aphasia, Syntactical

Syntactical Aphasia

Syntactical Aphasias

#1：(TS=(aphasia after stroke)) OR TS=(post-stroke aphasia)

#2：((TS=(Word Deafness)) OR TS=(Deafness, Word)) OR TS=(Alogia)) OR TS=(Alogias)) OR TS=(Anepia)) OR TS=(Anepias)) OR TS=(Logagnosia)) OR TS=(Logagnosias)) OR TS=(Logamnesia)) OR TS=(Logamnesias)) OR TS=(Logasthenia)) OR TS=(Logasthenias)) OR TS=(Aphasia, Acquired)) OR TS=(Acquired Aphasia)) OR TS=(Dysphasia)) OR TS=(Aphasia, Ageusic)) OR TS=(Ageusic Aphasia)) OR TS=(Ageusic Aphasias)) OR TS=(Aphasia, Auditory Discriminatory)) OR TS=(Auditory Discriminatory Aphasia)) OR TS=(Auditory Discriminatory Aphasias)) OR TS=(Discriminatory Aphasia, Auditory)) OR TS=(Discriminatory Aphasias, Auditory)) OR TS=(Aphasia, Commisural)) OR TS=(Aphasias, Commisural)) OR TS=(Commisural Aphasia)) OR TS=(Commisural Aphasias)) OR TS=(Aphasia, Functional)) OR TS=(Functional Aphasia)) OR TS=(Functional Aphasias)) OR TS=(Aphasia, Global)) OR TS=(Global Aphasia)) OR TS=(Global Aphasias)) OR TS=(Dysphasia, Global)) OR TS=(Global Dysphasia)) OR TS=(Global Dysphasias)) OR TS=(Aphasia, Graphomotor)) OR TS=(Graphomotor Aphasia)) OR TS=(Graphomotor Aphasias)) OR TS=(Aphasia, Intellectual)) OR TS=(Intellectual Aphasia)) OR TS=(Intellectual Aphasias)) OR TS=(Aphasia, Mixed)) OR TS=(Mixed Aphasia)) OR TS=(Mixed Aphasias)) OR TS=(Aphasia, Post-Ictal)) OR TS=(Aphasia, Post Ictal)) OR TS=(Post-Ictal Aphasia)) OR TS=(Post-Ictal Aphasias)) OR TS=(Aphasia, Post-Traumatic)) OR TS=(Aphasia, Post Traumatic)) OR TS=(Post-Traumatic Aphasia)) OR TS=(Post-Traumatic Aphasias)) OR TS=(Aphasia, Progressive)) OR TS=(Progressive Aphasia)) OR TS=(Progressive Aphasias)) OR TS=(Dejerine-Lichtheim Phenomenon)) OR TS=(Dejerine Lichtheim Phenomenon)) OR TS=(Phenomenon, Dejerine-Lichtheim)) OR TS=(Lichtheim's Sign)) OR TS=(Lichtheim Sign)) OR TS=(Lichtheims Sign)) OR TS=(Sign, Lichtheim's)) OR TS=(Aphasia, Semantic)) OR TS=(Semantic Aphasia)) OR TS=(Semantic Aphasias)) OR TS=(Aphasia, Syntactical)) OR TS=(Syntactical Aphasia)) OR TS=(Syntactical Aphasias)

#3：(((((((((((((((((((((((((((TS=(Strokes)) OR TS=(Cerebrovascular Accident)) OR TS=(Cerebrovascular Accidents)) OR TS=(Cerebral Stroke)) OR TS=(Cerebral Strokes)) OR TS=(Stroke, Cerebral)) OR TS=(Strokes, Cerebral)) OR TS=(Cerebrovascular Apoplexy)) OR TS=(Apoplexy, Cerebrovascular)) OR TS=(Vascular Accident, Brain)) OR TS=(Brain Vascular Accident)) OR TS=(Brain Vascular Accidents)) OR TS=(Vascular Accidents, Brain)) OR TS=(Cerebrovascular Stroke)) OR TS=(Cerebrovascular Strokes)) OR TS=(Stroke, Cerebrovascular)) OR TS=(Strokes, Cerebrovascular)) OR TS=(Apoplexy)) OR TS=(CVA (Cerebrovascular Accident))) OR TS=(CVAs (Cerebrovascular Accident))) OR TS=(Stroke, Acute)) OR TS=(Acute Stroke)) OR TS=(Acute Strokes)) OR TS=(Strokes, Acute)) OR TS=(Cerebrovascular Accident, Acute)) OR TS=(Acute Cerebrovascular Accident)) OR TS=(Acute Cerebrovascular Accidents)) OR TS=(Cerebrovascular Accidents, Acute)

#4： #2 AND #3

#5: #1 OR #4
